# The Prevalence of Toxoplasma gondii in Hemodialysis Patients

**DOI:** 10.5812/ircmj.5225

**Published:** 2013-10-05

**Authors:** Masomeh Bayani, Amrollah Mostafazadeh, Farsheed Oliaee, Narges Kalantari

**Affiliations:** 1 Department of Infectious Diseases and Tropical Research Center, Babol University of Medical Sciences, Babol, IR Iran; 2 Department of Microbiology-Immunology, Babol University of Medical Sciences, Babol, IR Iran; 3 Department of Internal Medicine, Babol University of Medical Sciences, Babol, IR Iran; 4 Cellular and Molecular Biology Research Center, Babol University of Medical Sciences, Babol, IR Iran

**Keywords:** Kidney Failure, Chronic, Renal Dialysis, Toxoplasma

*Dear Editor,*

Toxoplasma gondii (T. gondii) is an obligate intracellular protozoan parasite that can infect a wide range of hosts including humans. Humans are infected through raw or undercooked meat containing tissue cysts and ingesting oocysts through contaminated food, water supplies and gardening. Infection with T. gondii is characterized by acute and chronic phases, which are distinct with regards to the developmental stage of the parasite and the sites of parasite persistence (1). However, Toxoplasma infection is mostly asymptomatic in immunocompetent individuals but it may be life threatening in immunocompromised cases (2). There is some evidence that chronic renal failure patients undergoing hemodialysis are susceptible to acquire various infections (3). This study aimed to illustrate the clinical manifestations of toxoplasmosis in haemodialysis patients and seroconversion rates of T. gondii. 

The subjects were all haemodialysis patients admitted to the haemodialysis unit at Shahid Beheshti Hospital of Babol University of Medical Sciences, Babol, northern Iran and 50 healthy volunteers. Individuals with normal creatinine, blood urea nitrogen (BUN) and without any renal disease were randomly enrolled in this study. In addition to demographic data, period of haemodialysis treatment and any manifestation of acute toxoplasmosis such as fever of unknown origin, lymphadenopathy and encephalitis were recorded. Two milliliters blood was taken from each participants, serum was separated and then the existence of anti-T. gondii antibodies, IgG and IgM were measured using a commercial enzyme-linked Immunosorbent assay (ELISA) kit (Trinity Biotech Captia, USA). The tests were carried out according to the manufacture's recommendation. Five out of 18 seronegative cases were died and three out of 18 seronegative cases received renal transplant. One seronegative case of the healthy volunteer was not participated in the second experiment. The data were analyzed by t-test and Fisher's test.

The average age of patients and the control group was 42.5 ± 11.5 and 34.8 ± 10.4, respectively. Fifty four out of 90 (60%) haemodialysis patients and 20 out of 50 (40%) healthy volunteers were female. The mean duration of dialysis was 5 ± 4.5 years. Seventy two out of 90 (80%) patients and 38 out of 50 (76%) healthy voluntaries were positive for Anti-T. gondii (IgG). No statistically significant difference was found between two groups (P > 0.05). IgM antibodies were not found in any of the cases among both groups in the first series of the experiment. In the second experiment which was carried out on seronegative cases, IgG and IgM, were detected in eight and two out of ten serum samples among haemodialysis patients, while IgG antibodies were detected in one serum sample within healthy volunteer group. There was a significantly higher seroconversion rate in chronic renal failure patients group and healthy volunteer group (80% versus 11.1%) (P = 0.002). Data obtained from the assessment of haemodialysis treatment duration and having anti-T. gondii antibodies showed that no relationship was seen between period of haemodialysis and the rate of seropositivity (P = 0.961). The demographic information data are presented in Table 1. Regular physical examination of haemodialysis patients did not demonstrate any manifestation of acute Toxoplasma infection due to reactivation of the parasite or recent infection. 

**Table 1.** Gender and Residency-Related Prevalence of IgG Antibodies to Toxoplasma Gondii in Haemodialysis Patient and Healthy Volunteers Groups

Groups (variable)	Haemodialysis Patients Group, No. (%)		Healthy volunteer Group, No. (%)	
	Positive	Negative	Positive	Negative
**Gender**				
Female	46 (51.1%)	8 (8.9%)	13 (26%)	7 (14%)
Male	26 (28.9%)	10 (11.1%)	25 (50%)	5 (10%)
Total	72 (80%)	18 (20%)	38	12
**Residency**				12 (24%)
Urban	33 (36.7%)	18 (20%	30 (60%)	0 (0%)
Rural	29 (32.3%)	10 (11%)	8 (16%)	
Total	72	28	38	12

The findings obtained from the current study demonstrated a high rate of T. gondii infection in haemodialysis patients (80%) and healthy volunteers (76%), in comparision with the other studies (4). Risk factor variety predisposing to Toxoplasma infection such as age, climate conditions, agriculture activity, occupation and eating habits are the possible explanations for these differences (5, 6). Furthermore, assessment of anti-Toxoplasma antibodies in seronegative cases after one year showed that haemodialysis patients are at risk of infection either due to recent infection or reactivation of the parasite. Moreover, the percentage of seropositivity among females was significantly higher in the patient group but it was reverse in control group. Some researchers did not find a significant relationship between Toxoplasma infection rate and gender (7) whereas some other studies found it (6). Different sampling methods, diverse life styles, or gender differences in different communities could explain this diversity. Also, a non-significant difference was seen between seropositivity of patients who lived in rural areas compared with urban areas. On the other hand, this study did not demonstrate any manifestation of toxoplasmosis in the patients during regular physical examinations throughout a year. Nonspecific symptoms in toxoplasmosis e.g. fever is a possible reason for misdiagnosis or forgotten the Toxoplasma infection (8). Another possibility is the production of same cytokines in Toxoplasma infection and haemodialysis patients such as Interleukin 12 (IL-12) which plays a major anti-Toxoplasma role during the acute phase of the infection. Also, production of IL-2, IL-4 and IL-10 which are required for the development of resistance to the infection was increased in haemodialysis patients (9, 10). Therefore, this study does not recommend regular serological tests for haemodialysis patients except who are candidate for kidney transplant.
